# Psychiatric epidemiology and the Chicago School of Sociology

**DOI:** 10.1177/0957154X231206510

**Published:** 2023-12-06

**Authors:** Matthew Smith

**Affiliations:** University of Strathclyde, UK

**Keywords:** Chicago, homelessness, mental illness, psychiatric epidemiology, sociology, suicide

## Abstract

This article explores the Chicago School of Sociology’s influence on psychiatric epidemiology. While the Chicago School text usually associated with psychiatric epidemiology is the 1939 book by Faris and Dunham, it is important to acknowledge the influence of earlier Chicago School projects during the 1920s. These projects, tackling everything from homelessness and delinquency to the ghetto and suicide, provided models not only for Faris and Dunham, but also for numerous methodological and theoretical insights for the social psychiatry projects that would emerge after World War II. The social sciences and the humanities still have important roles to play in informing contemporary approaches to psychiatric epidemiology and deriving ways to tackle the socio-economic problems that contribute to mental illness.

## Introduction

In 1939 Robert EL Faris (1907–98) and H Warren Dunham (1906–85) published *Mental Disorders in Urban Areas: An Ecological Study of Schizophrenia and Other Psychoses*, one of the first large-scale works to address the epidemiology of mental illness in a US city. Based largely on their research as graduate students at the University of Chicago’s famed Chicago School of Sociology, the study used admissions statistics from Cook County’s psychiatric hospitals to determine where patients had lived prior to admission, and they then hypothesised about the relationship between the urban environment and the epidemiology of different mental disorders. Its conclusion was that people who lived in communities marked by social isolation, community disintegration and poverty were more likely to experience mental distress and be hospitalised. In so doing, Faris and Dunham contributed to the broader project initiated by anthropologist Franz Boas (1858–1942) to undermine the previously dominant hereditary explanations for mental illness and to set the tone for the social psychiatry research that would flourish following World War II (Smith, 2023). Beginning in the late 1940s, a series of interdisciplinary social psychiatry projects would follow, many funded by the newly-founded National Institute of Mental Health. These studies, which concentrated on psychiatric epidemiology, provided an evidence base for the emergence of the community mental health movement in the USA and, specifically, the passage of the Community Mental Health Act of 1963, which accelerated the shift away from the asylum and to community-based mental health care (Hollingshead and Redlich, 1958; Leighton, 1959; Srole et al., 1962).

It can be argued, therefore, that *Mental Disorders in Urban Areas*, a work of sociology informed by urban geography, served as an important catalyst for monumental changes in US mental health policy during the second half of the twentieth century. The role of the Chicago School has been mentioned briefly in some accounts of the history of psychiatric epidemiology, but it has not been addressed in detail (Grob, 1985; Lovell and Susser, 2014; March and Oppenheimer, 2014). Moreover, the social psychiatry projects that would emerge after World War I in the USA also situated the social sciences, chiefly sociology and anthropology, at the very heart of their research, as historians have noted (Campbell, 2014; Demazeux, 2014; March and Oppenheimer, 2014; Scull, 2011a, 2011b; Smith, 2023). Most such projects were either collaborations between social scientists and psychiatrists, such as August B Hollingshead and Frederick C Redlich’s *Social Class and Mental Illness* (1958), which focused on New Haven, Connecticut, or in the case of the Stirling County Study, were led by researchers, such as Dorothea and Alexander Leighton (Hughes et al., 1960; Leighton, 1959; Leighton et al., 1963), who were trained in both psychiatry and the social sciences. In what follows, I trace the intellectual origins of *Mental Disorders in Urban Areas* (Faris and Dunham, 1939) and its approach to psychiatric epidemiology by exploring how mental health and related topics were researched in the Chicago School during the 1920s. I begin by discussing how Chicago School researchers investigated other social problems, such as homelessness, ghettoisation and delinquency, and then turn to Ruth Shonle Cavan’s *Suicide* (1928), which had the strongest influence on Faris and Dunham’s research in terms of both its topic and its approach. In so doing, I highlight the distinct, if sometimes indirect, influence these early works of sociology had, not only on psychiatric epidemiology, but also on how mental health services in the USA would evolve after World War II.^1^ I conclude by arguing that today it is just as important for researchers in the social sciences and humanities to inform psychiatric epidemiology, and mental health services and mental health policy more generally.

## Introducing the Chicago School of Sociology

The University of Chicago’s Department of Sociology dates back to 1892 and the foundation of the university itself.^2^ As with all of the first US sociology departments, it was influenced by early European sociologists, such as Émile Durkheim (1858–1917) and Max Weber (1864–1920). Although the subject had been taught elsewhere in the USA prior to 1892, the University of Chicago’s department was the first such unit dedicated solely to sociology and it would become ‘the single main source of both the intellectual and institutional development of the field, including research on the sociology of medicine’ (Bloom, 2002: 63). Unlike the nascent sociological investigations that had been undertaken in Britain by reformers and writers, such as Beatrice Webb (1858–1943), the Chicago School’s approach was meant to be objective and scientific, rather than to achieve political ends. As Robert Faris (1967/1970) stated in his own insider’s account of the department between 1920 and 1932, previous British research was ‘hopelessly emotional. It could, and sometimes did, lead to political action but not towards science; rather, it tended to draw the participants away from science’ (p. 7). According to Samuel W Bloom (2002: 24), this ‘tension between advocacy and objectivity, between applied and basic science’ was present early in the development of sociology and would continue to loom large. Although debates would emerge within the Chicago School about the degree to which sociologists should remain detached from politics and activism, one of its chief goals was to establish sociology as an objective social science (Carey, 1975).

The city of Chicago itself proved to be the ideal laboratory for the Chicago School. Following the Great Fire of 1871, Chicago quickly changed from a ramshackle trading post to a modern metropolis, boasting the world’s first skyscrapers. One key development in this transformation was creating a central business district that saw the downtown core designated primarily for commercial, rather than residential, use. Also influential was the influx of Black migrants from the American South and also European immigrants, which contributed to rapid population increases. While these changes resulted in economic growth, alongside them emerged entrenched social problems, ranging from racial tension and desperate poverty to notorious gangland crime and vice. All these issues would become topics of interest for the Chicago School.

However, few of the first generation of Chicago School sociologists concentrated significantly on the study of health and illness. One exception can be found in the work of WI Thomas (1863–1947), whose first book, *Sex and Society: Studies in the Social Psychology of Sex* (1907), was, as its title indicated, rooted in the nascent field of social psychology. Social psychologists may not have focused specifically on health, but they often addressed issues, such as social deviance, that resonated with those interested in mental illness. Thomas, with Florian Znaniecki (1882–1958), also wrote *The Polish Peasant in Europe and America* (1918–20), arguably the most innovative and influential work to be produced by members of the first generation of the Chicago School, which introduced the use of life histories to sociological research.^3^ By the 1920s, however, when the second generation of the Chicago School came into its ascendency, health and illness would emerge as a more popular topic for both established researchers and graduate students. One catalyst for this was the appointment of Ellsworth Faris (1874–1953), father of Robert Faris, as chair of Chicago’s Department of Sociology and Anthropology, replacing the founding chairperson Albion Small (1854–1926) when he retired in 1923.^4^ The elder Faris had served as a missionary in the Congo between 1897 and 1904, which triggered his interest in the social sciences. In his teaching, he would challenge the ideas of Herbert Spencer, Lucien Lévy-Bruhl and Sigmund Freud regarding the nature of so-called ‘primitive man’, arguing that the term ‘preliterate’ was preferable (Anon., 1954). After he was invalided home, he began a doctorate in psychology, specialising in social psychology. His appointment to the chair of Sociology and Anthropology was intended in part to serve as a bridge between his department and that of psychology (Odum, 1951: 181–5). It was also hoped that he would be able to revitalise and rebuild the department since it had recently lost all of its pioneering members except Small, who was struggling with a heart condition. He also undoubtedly influenced the research of his son, Robert, on psychiatric epidemiology.

Two of the sociologists who aided Ellsworth Faris in his task were Robert E Park (1864–1944) and Ernest W Burgess (1886–1966).^5^ Park came to sociology late in life, as had many early sociologists. But while many of Park’s predecessors had trained for the ministry, he had worked as a reporter for 11 years, writing muckraking stories about squalor, corruption and criminality in urban America (Bovenkerk, 2010). After earning his PhD in 1903 at the age of 39, he worked for Booker T Washington (1856–1915) at the Tuskegee Institute for another seven years, serving as the Black educator and civil rights leader’s secretary and ghostwriter. He finally came to the University of Chicago at the age of 50, having been invited by Thomas. Burgess, originally from Canada, had a more direct path to his sociological career, coming to Chicago for graduate studies and completing his PhD in 1913. He, along with Louis Wirth (1897–1952), was listed as an advisor for Faris and Dunham’s project (Faris and Dunham, 1939: 11). Together, Park and Burgess would write two highly influential texts. The first was *Introduction to the Science of Sociology* (1921), which, according to Odum (1951: 169), ‘was the most influential sociology text of any that had been written and perhaps of any that has yet been published’.

**Figure 1.**
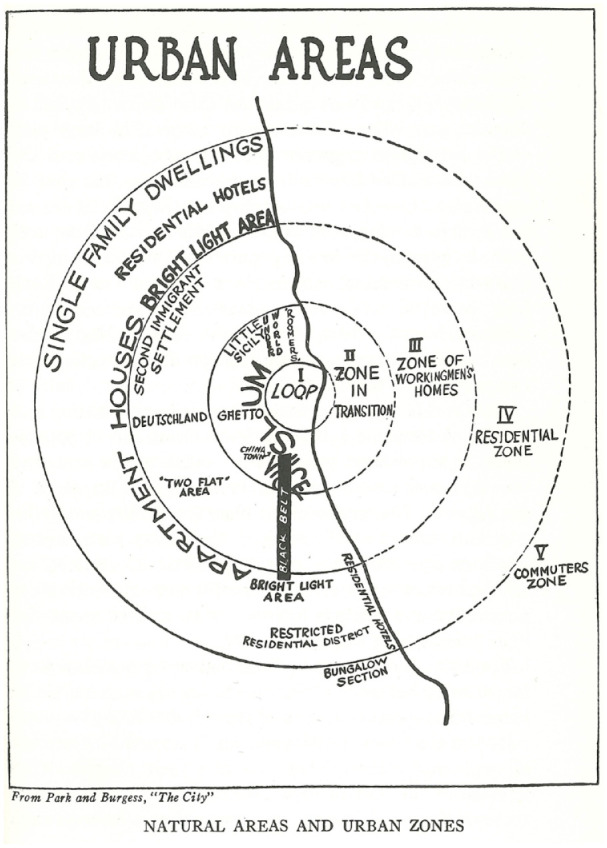
Model of Ernest W Burgess’s Concentric Zone Theory (Park and Burgess, 1925: 55).

**Figure 2.**
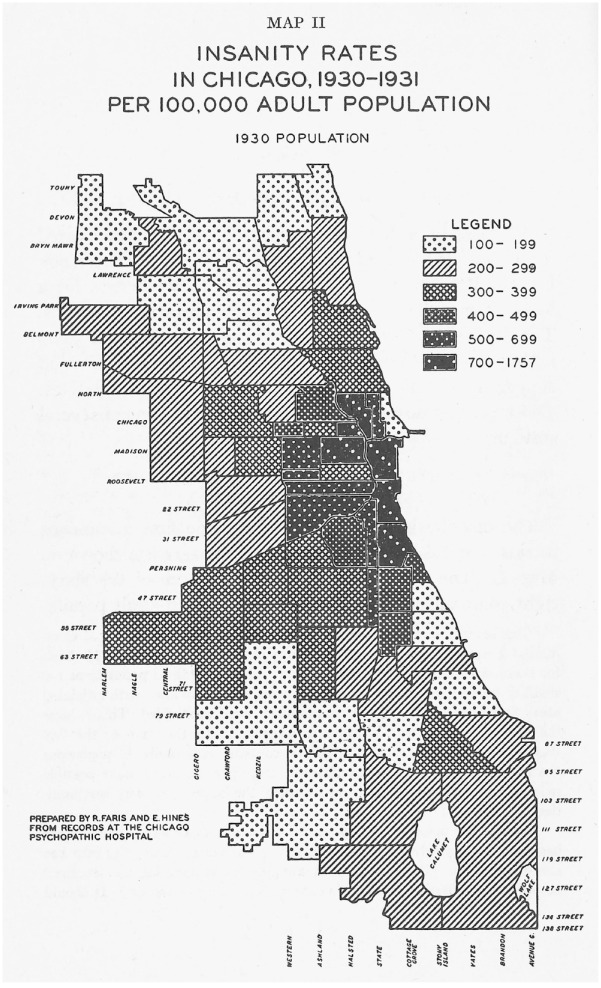
Map of insanity rates in Chicago, 1930–31 (prepared by R Faris and E Hines from records at the Chicago Psychopathic Hospital).

**Figure 3.**
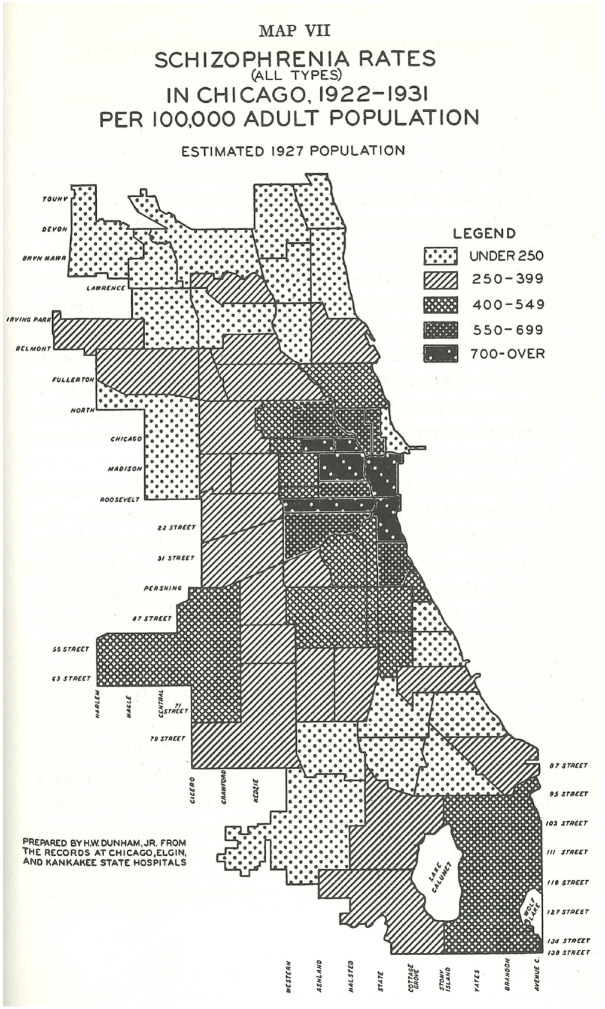
Map of schizophrenia rates in Chicago, 1922–31 (prepared by HW Dunham Jr from records at Chicago, Elgin and Kankakee State Hospitals).

**Figure 4.**
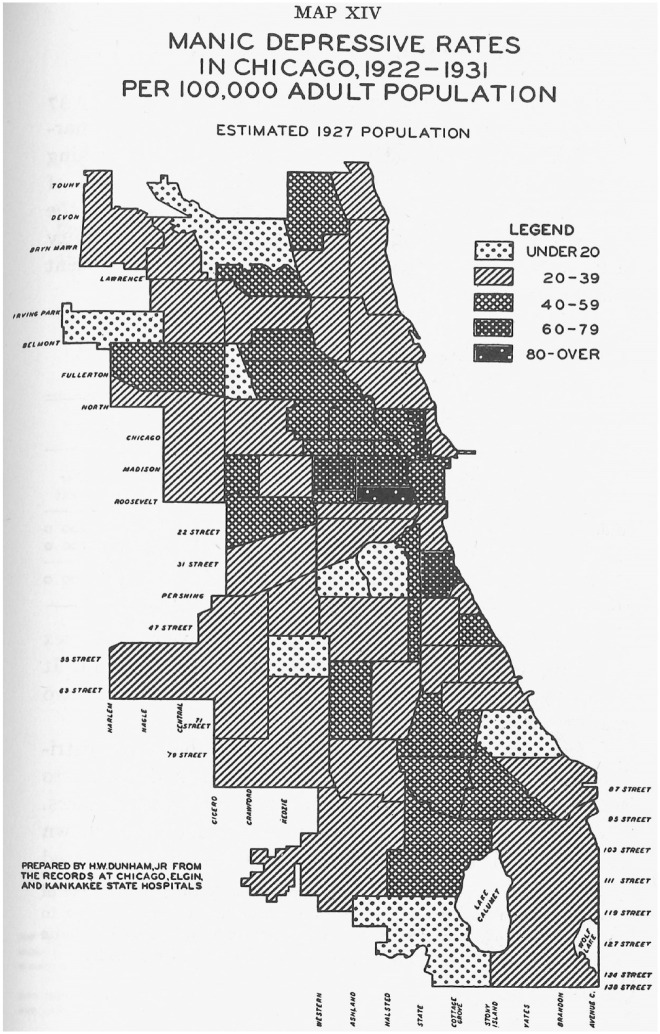
Map of manic-depressive rates in Chicago, 1922–31 (prepared by HW Dunham Jr from records at Chicago, Elgin and Kankakee State Hospitals).

In addition to this ‘bible of sociology’, the pair also compiled *The City* (Park and Burgess, 1925), a collection of their own essays and also contributions by Chicago School members Roderick D Mackenzie (1885–1940) and Louis Wirth. The first two chapters in particular, written by Park and Burgess respectively, helped to provide a theoretical and contextual framework for Faris and Dunham’s *Mental Disorders in Urban Areas*, but in different ways. Park’s essay ‘The city: suggestions for the investigation of human behavior in the urban environment’, for instance, emphasised how the city was not ‘merely a physical mechanism and an artificial construction’ (Park, 1925: 1). Rather, it was ‘a product of nature, and particularly of human nature’, and existed as ‘a state of mind, a body of customs and traditions, and of the organized attitudes and sentiments that inhere in these customs’ (p. 1). Park proceeded to emphasise the complex nature of city life, listing dozens of questions throughout the chapter that could frame a panoply of sociological investigations. The chapter that followed, by Burgess, helped Faris and Dunham to identify their specific inquiry, namely, the relationship between place of residency and mental disorder. Burgess’s essay ‘The growth of the city’, as its title indicated, explored how cities grew, but also how such growth also resulted in deterioration. Using Chicago as his model, Burgess described how, as cities expanded, they sifted and sorted ‘individuals and groups by residence and occupation’, creating a series of concentric zones that were characterised by certain types of residents, ranging from homeless migrants (hobos) and impoverished immigrants to skilled factory workers and business people. Burgess divided Chicago into five zones, radiating out from the central business district or ‘Loop’ (Burgess, 1925). Social problems tended to concentrate in the inner zones. Since these inner zones tended to be dominated by commercial developments, the only residents were usually transient individuals, including the city’s many homeless in Hobohemia, the subject of Nels Anderson’s research (see below). Many Chicago School researchers, including Faris and Dunham, used Burgess’s theory as a framework for their own studies. While Burgess illustrated his concentric zone theory in the abstract (see Figure 1), Faris and Dunham employed dozens of maps to show the distribution of various mental disorders in Chicago. Overall, Faris and Dunham found that Burgess’s concentric zone theory provided a compelling explanation for the distribution of mental disorders. Mental disorders as a whole tended to be concentrated in the most impoverished, transient and disintegrated regions near Chicago’s central business district (Figure 2).^6^ The pattern was even clearer for schizophrenia (Figure 3), but broke down considerably for manic depression (Figure 4), which was more evenly distributed throughout the city. Such differences suggested that the epidemiology of some mental disorders was more determined by socio-economic factors than others.

## Hobos and ghetto dwellers

While Park and Burgess’s *Introduction to the Science of Sociology* and *The City* helped to establish the Chicago School as the pre-eminent sociology department in the USA, it is arguable that their supervisory impact was equally significant. Most of the Chicago School sociologists who came to prominence during the 1920s and 1930s were supervised by Park and/or Burgess (as well as Ellsworth Faris), including those who influenced Faris and Dunham. The urban environment, and Chicago in particular, became a popular topic. The first of a long series of graduate students to produce a field research monograph in this tradition was Nels Anderson (1889–1986), who published *The Hobo: The Sociology of the Homeless Man* in 1923, two years before he finished his Master’s degree at the University of Chicago (he would later complete his PhD at New York University).

Anderson was more qualified than most to research his topic. The son of Swedish immigrants, he had grown up in a variety of unconventional settings all over the USA, including logging, road construction and mining camps, farms, a Nez Perce reservation, and the slums of Chicago. After high school Anderson wandered the western USA as an itinerant hobo, working as a ‘newsboy, mule-skinner, mine worker, track repairman, coal forker, field hand, railroad maintenance carpenter, timberman, grade school teacher, concrete former, millwright, Army engineer and demolitions experts, itinerant peddler, and male nurse’ (Iverson, 2009: 181). He rode the rails, found himself penniless on occasion and had to panhandle or beg for food.^7^ He began his studies at Brigham Young University in Utah before proceeding to the University of Chicago for graduate studies. Anderson quickly began researching the hobo life that he knew so well and, with the support of the Chicago Council of Social Agencies and the Laura Spelman Rockefeller Fund, began researching and writing *The Hobo*.^8^

Anderson’s highly novel use of participant observation and life-story interviewing was one of the reasons why *The Hobo* became so influential.^9^ Along with countless other social science studies, many social psychiatry projects of the 1950s, most notably Alexander and Dorothea Leighton’s Stirling County Study and August Hollingshead and Frederick Redlich’s New Haven Study, would also employ participant observation and extensive interviewing (Smith, 2023: chs. 3; 5). But for Faris and Dunham, who primarily used quantitative methods, the book’s most influential element was one of its core research questions, specifically, were hobos born or made? In other words, were individuals predisposed by genetic inheritance to live the unstable, transient life of a hobo or were hobos the product of social, cultural and economic factors? Faris and Dunham’s study would ask the same question regarding mental disorder.

Ultimately, both studies would emphasise the role of environmental factors as opposed to heredity, an argument that many Chicago School researchers had made regarding other social problems (Chapoulie, 2001/2020: 56). Although each study employed different methodologies, they both revealed complexities with respect to the environmental origins of both the hobo and the mentally disordered. For Faris and Dunham, a puzzle emerged when they compared the geographical distribution of schizophrenia with that of manic depression, as described above. For Anderson (1923: 61), questions about what led an individual to become a hobo ‘invariably raised other questions even more difficult to answer’. Overall, he identified six main factors, specifically: seasonal work and unemployment, industrial inadequacy, defects of personality, crises in the life of the person, racial or national discrimination, and wanderlust. Within each category, however, could be another layer of underlying explanations. Anderson saw industrial inadequacy, or an inability to hold down a job in the fast-paced environment of industrial work, as being a problem in particular for those who were either ‘feebleminded’ or ‘emotionally unstable’ (p. 65). But others struggled in industry because of occupational injuries (ranging from amputations to lung disease), addictions and old age. It was impossible, Anderson argued, to identify a single cause ‘to explain how a man may be reduced to the status of a homeless, migratory, and casual laborer’ (p. 85). Moreover – and foreshadowing discussions of intersectionality 100 years later – Anderson suspected that these factors combined in the cases of many hobos. Despite these complexities, however, he believed that remedial and preventive approaches to homelessness were possible so long as one recognised that the root causes were:

. . . at the very core of our American life, in our industrial system, in education, cultural and vocational, in family relations, in the problems of racial and immigrant adjustment, and in the opportunity offered or denied by society for the expression of the wishes of the person. (p. 86)^10^

Although Faris and Dunham were more reluctant to implicate such issues in the epidemiology of mental disorder, Anderson’s portrayal of hobo life and of Hobohemia was nevertheless woven into their book *Mental Disorders in Urban Areas*. Their description of Hobohemia may not have contained the rich case studies found in *The Hobo*, but it still reflected what Anderson had written about its characteristics as well as those who lived there. Hobohemia’s residents were ‘the most unstable in the city’ and ‘extremely isolated’, resorting to thievery, begging, charity, the bottle and sexual perversions, living lives ‘without goal or plan’, drifting ‘aimlessly and alone, always farther from the conventional and normal ways of living’ (Faris and Dunham, 1939: 5–6). What Faris and Dunham’s research added to this picture was that the denizens of Hobohemia were also the most likely residents of the city to become hospitalised due to schizophrenia.

Faris and Dunham’s understanding of marginalised urban spaces and features of Chicago’s ethnically and racially segregated neighbourhoods was also influenced by another recent doctoral graduate, Louis Wirth, who served as one of their project advisors. Wirth was born in Germany and immigrated to the USA, ending up in Omaha, Nebraska, in 1911, when he was 14 years old. Three years later he was studying at the University of Chicago with Park, Burgess and others, completing his PhD in 1926. His first monograph, *The Ghetto* (Wirth, 1928), was based on his doctoral research (supervised by Park), and combined a broader discussion of the origins of the Jewish ghetto in Europe with a detailed analysis of Chicago’s ghetto.

Wirth’s research informed Faris and Dunham’s understanding of community integration and, in turn, the perils of community disintegration, a theme that would resonate strongly in the Stirling County Study of Dorothea and Alexander Leighton, as well as other social psychiatry studies. Chicago’s Jewish ghetto was located in a densely populated area of the city ‘[w]est of the Chicago River, in the shadow of the Loop’ that contained most of the city’s immigrant communities and, according to Wirth (1928: 195), represented ‘the most varied assortment of people to be found in any similar area of the world’. When these immigrant groups settled, they initially maintained their language, customs and institutions, contributing to social control and little community disintegration. This, as Faris and Dunham noted, was particularly true of the Russian-Jewish ghetto described by Wirth, where ‘Old-World cultures are preserved almost intact’ (Faris and Dunham, 1939: 8). When these traditions began to erode (due to the influx of individuals from different cultural backgrounds and intergenerational conflict), however, the social cohesion of these communities rapidly disintegrated, leading to ‘extreme poverty’ and ‘high rates of juvenile delinquency, family disorganization, and alcoholism’ (p. 8). As Wirth (1927: 71) described, the ghetto, whether it be ‘Chinese, Negro, Sicilian, or Jewish’ was ‘not merely a physical fact, but also a state of mind’.

Similarly, E Franklin Frazier’s *The Negro Family in Chicago* (1932) also emphasised the potentially pathological impact of migration in the case of Black Americans who came to urban centres from the American South. While there are debates about the extent to which the work of Frazier (himself the son of freed slaves) was imbued with the idea of Black pathology, thus according with the prevailing racist attitudes of the time (Jelks and Hardison, 2019; Platt, 1993), he also acknowledged how the transition from the familiar, yet coercive, environment of the rural South to ‘the stern competition of city life’ presented unique stresses for Black Americans (Frazier, 1932: 73). Migrating to a city could trigger a ‘crisis’, due in part to the disintegration of family and friendship groups and possibly the loss of relative status some Black Americans enjoyed within their communities (p. 74).

Faris and Dunham’s findings regarding race/ethnicity and mental health echoed some of the insights of both Wirth and Frazier, but with some interesting caveats. Overall, no race or ethnicity was more likely to be hospitalised for mental disorder than any other, although the rates for specific disorders varied somewhat according to ethnicity. What Faris and Dunham did find, however, was that mental illness was highest in Black Chicagoans who lived in areas such as the Loop, and *not* in predominantly Black communities, for example, the so-called ‘Black Belt’. The same tendency occurred for members of other migrant groups. In turn, both native-born and foreign-born white Chicagoans had higher rates of mental illness if they lived in Black communities. These findings, along with those of psychiatric statistician Benjamin Malzberg (1936), helped to undermine some of the racist assumptions regarding mental illness that were predominant during this period (Doyle, 2016; Fielder, 2022; Hoberman, 2012; Rose, 2009; Stewart, 2013). They also strengthened the idea that the degree to which an individual was either integrated into or isolated from their community was a key factor in mental health outcomes. In some cases, this was because an isolated individual struggled to fit into an otherwise integrated community. But in disintegrated communities, such as Hobohemia or the Loop, all residents were at risk of such isolation.

The research of Wirth, Frazier and other Chicago School sociologists also contributed to broader debates about whether cities were good or bad for mental health. Previous sociologists had suggested that, while the anonymity of city life could be liberating, it could also leave people feeling detached, alone and unimportant (Simmel, 1903/2012). Frazier’s research revealed that similar dynamics applied to Black migrants to Chicago. On the one hand, migrants might benefit psychologically from being freed from the scrutiny of the church and of local gossip. But on the other hand, institutions, such as the church, provided structure, sociability and security. Without them, migrants could become disillusioned and detached. Although Faris and Dunham did not address these debates about cities and mental health in great detail, the issue loomed large in subsequent social psychiatry studies (Pols, 2003). The Midtown Manhattan Study, in particular, was designed in part to understand the relationship between cities and mental health. Its startling findings, that 81.5 per cent of Manhattanites had some symptoms of mental illness, made headline news (Srole et al., 1962: 138). What the newspapers did not acknowledge, however, was that the Stirling County Study of rural Nova Scotia also found similar rates of mental illness. It was not cities *per se* that were bad for mental health, but rather factors such as poverty, inequality, social isolation and community disintegration – and these could be found in both urban and rural settings (Pols, 2003; Smith, 2023: ch. 4).

Such nuances, however, were often lost in the efforts to clear slums after World War II, as the research of Jane Jacobs (1961), Herbert Gans (1962), Erich Lindemann and others indicated (Ramsden and Smith, 2018). In 1958, for instance, the Boston Redevelopment Authority began demolishing a 48-acre section of Boston’s West End that had been home to 2700 families, including a large number of lower-working-class Italian-Americans. One of its rationales was that the neighbourhood was considered to be an unhealthy space that was conducive to mental illness and other social problems. However, research led by social psychiatrist Erich Lindemann (1900–74), sociologist Herbert Gans (b. 1927) and social psychologist Marc Fried (1922–2008) on the West End prior to its demolition, suggested that many residents found it to be a good place to live and certainly not a slum. If anything, the prospect of their neighbourhood’s destruction had worsened the mental health of many residents, leaving many depressed and a few suicidal (Fried, 1966).

## Mapping delinquency and suicide

Although Faris and Dunham were influenced by the conclusions made by Wirth, Frazier and Anderson, they did not emphasise the qualitative methods used by these sociologists, namely, interviewing, participant observation and the application of historical analysis. Instead, their research was primarily quantitative, using statistics from hospital admissions and illustrating the correlation between psychiatric epidemiology and urban geography using maps. Here, their approach was more informed by Clifford Shaw’s (1895–1957) *Delinquency Areas* (1929) and Ruth Shonle Cavan’s (1896–1993) *Suicide* (1928).^11^ While both Shaw and Cavan used qualitative methods extensively, they also employed maps to show how delinquency and suicide were each associated with the different zones of the city identified by Burgess. This geographical approach would provide the clearest model for Faris and Dunham of the best approach to psychiatric epidemiology.

Clifford Shaw began graduate studies at the University of Chicago in 1919, after finishing his undergraduate degree at Adrian College in Michigan. During his doctoral training, which he did not finish, he worked part time as a parole officer and a probation officer for the Cook County Juvenile Court, roles which put him in close contact with young people involved in the justice system, their family and community backgrounds and the court system (Gelsthrope, 2010). He would also become the director of the Institute for Juvenile Research in 1926, the year in which the research that led to *Delinquency Areas* (Shaw, 1929) began in earnest.

Shaw’s *Delinquency Areas* and Faris and Dunham’s *Mental Disorders in Urban Areas* share one particular feature in common: each book contains dozens of maps, charting the distribution of juvenile delinquency and mental disorder, respectively. In this way, both projects owed a debt to Ernest Burgess and his concentric zone theory. With respect to delinquency, Burgess described how, in the ‘zone in transition . . . play is crime . . . . Boys’ gangs, juvenile delinquency, poverty, desertion, bad housing abound’ (Burgess, quoted in Shaw, 1929: 19–20). He proceeded to explain that such problems dissipated towards the outer zones and the suburbs. Shaw’s research bore this out, as did that of Chicago School sociologist Frederic Thrasher (1892–1962), whose work focused on gangs (Thrasher, 1927). Based on the stated residences of 55,000 delinquents (predominantly juvenile delinquents, but also truants and some adult offenders) who went through the Cook County courts, Shaw (1929: 33) demonstrated that delinquents were more likely to live in the deteriorated regions toward the centre of the city. These were, therefore, Shaw’s ‘delinquency areas’. This mirrored what Faris and Dunham would find with respect to mental disorder as a whole, though the distribution of manic depressive disorder was less concentrated in these central regions than that of most types of schizophrenia.

But the maps were only one part of Shaw’s analysis. In other ways, Shaw’s pioneering life history work with juvenile delinquents and the way in which he applied the findings of his research departed significantly from Faris and Dunham’s methodological approach and their willingness to apply the findings of their research beyond academia. In between the maps were extensive life histories of some of the boys and girls who made up the statistics. Although Faris did conduct some life histories (Faris, 1944), they did not feature substantially in *Mental Disorders in Urban Areas* (Faris and Dunham, 1939). Shaw gathered such stories by persuading young people in correctional institutions to write their own biographies (Gelsthrope, 2010). In addition to featuring in Shaw’s *Delinquency Areas*, he published some of these life histories, including that of ‘Stanley’, in *The Jack Roller: A Delinquent Boy’s Own Story* (1930). Shaw’s pioneering approach to life histories would not only inspire others within the Chicago School, but would also inform the work of some social psychiatrists, most notably Dorothea and Alexandre Leighton and their Stirling County Study (Leighton, 1959). During an era when psychoanalytic approaches to psychiatry were dominant in the USA, it was somewhat appropriate that the life histories of individuals were also feeding into ideas about psychiatric epidemiology.

Shaw’s use of life histories cemented his view that the social environment was all important in explaining delinquency. When interpreting the geographical distribution presented in their maps, Faris and Dunham followed Shaw’s lead:

high rates of delinquency were products not of the biological inferiority of the population stocks that inhabit the slum areas, not of any racial or national peculiarity, but rather of the nature of the social life in the areas themselves. (Faris and Dunham, 1939: 20)^12^

Specifically, delinquency areas were those characterised by being in a ‘process of transition from residence to business and industry and are characterized by physical deterioration, decreasing population, and the disintegration of the conventional neighbourhood culture and organization’ (Shaw, 1929: 204). The influx of migrants (both Europeans and Black Americans from the South) exacerbated the situation since their cultural mores and traditions tended to break down in these deteriorating neighbourhoods. It is important here to note how Shaw stressed the role of this ‘process of transition’ and the resulting disintegration, rather than the other social problems present in such areas:

It has been quite common in discussions of delinquency to attribute causal significance to such conditions as poor housing, overcrowding, low living standards, low educational standards, and so on. But these conditions themselves probably reflect a type of community life. By treating them one treats only symptoms of more basic processes. (p. 205)

In other words, the root cause of delinquency was social disintegration triggered by the ‘process of transition’, rather than the more direct influence of other socio-economic factors, such as poverty, inequality or poor housing. Poverty, for instance, was seen as a ‘symptom’ rather than a ‘basic process’ or ‘causal’ factor. This emphasis away from such social factors may have been subtle, but it nevertheless undermined more empowering and bottom-up approaches to reversing social disintegration and preventive mental health (for example, through enhanced welfare programmes or income supplementation) in favour of more top-down methods (such as slum clearances or community education programmes).

Regardless of what he saw as the root cause for delinquency, Clifford Shaw was nevertheless very active in tackling the problem directly. In the debates that Chicago School sociologists had about ‘advocacy’ and ‘objectivity’, he was very much in the former camp, along with Burgess and Wirth, who advocated that sociologists should engage with ‘clinical sociology’ and support the work of child guidance and mental hygiene (Wirth, 1931). In 1934, for example, Shaw’s research on delinquency led him to found the Chicago Area Project, which he would direct for the remainder of his life. This project, which still operates today, was founded to integrate disintegrated communities in Chicago, such as Russell Square in South Chicago, by forming ‘community committees’ within these neighbourhoods to work with predelinquent and delinquent groups. Central to its work was involving community members, including the delinquents themselves, in planning and running activities, ranging from basketball leagues to study clubs (Schlossman and Sedlak, 1983).

Faris and Dunham, in contrast, were much more on the ‘objectivity’ side of the debate, along with Park. Although Dunham had worked as a social worker with homeless men during his graduate studies, he approached psychiatric epidemiology from a markedly detached and academic outlook (Dunham, 1986). According to Faris’s obituary, written by his son who would also become a sociologist, ‘an essential element of the discipline of sociology was keeping it as clear as possible of emotion and politics’, which could cause the ‘intellectual contamination’ of sociology (Faris, 1998). In turn, Faris and Dunham’s *Mental Disorders in Urban Areas* did not contain much in the way of policy recommendations. They believed that their research had confirmed their hypothesis that disorganised communities led to social isolation, which in turn was associated with schizophrenia, but they said nothing about what should be done about it. Indeed, the only hint of how to apply their findings was provided by Ernest Burgess, who, as described above, believed that the work of sociologists should transcend the academy and, as one of the doctoral advisors of both researchers, wrote the introduction to the book. In it, he declared that:

[i]f social conditions are actually precipitating factors in causation, control of conditions making for stress and strain in industry and society will become a chief objective of a constructive program of mental hygiene. . . . Local community programs of mental hygiene can accordingly be directed to dealing with the indirect and precipitating causes of specific causes of psychoses, i.e., syphilis, as in the case of dementia paralytica, or lack of social contacts as with the young precatonic. (Burgess, 1939: xvii)

Faris and Dunham may not have expanded on Burgess’s ideas about how to apply their findings through mental hygiene initiatives. But their work, inspired by Shaw and others, did help to confirm a broader point: that mental illness – as with delinquency – had a social dimension and that it should not be considered in biological or neurological terms alone.

Of all the Chicago School sociologists, however, the one who had the most profound influence on Faris and Dunham in terms of both their topic and approach was Ruth Shonle Cavan and her monograph *Suicide* (1928). Her research, although only briefly referred to twice in *Mental Disorders in Urban Areas*, provided the inspiration for Faris and Dunham. Specifically, as Faris’s son recalled in the obituary he wrote for his father, Robert Park asked Robert Faris about what he would like to research for his doctoral dissertation. Faris replied that he had been impressed the way in which Cavan (then Ruth Shonle) had presented the distribution of suicide in Chicago. Park immediately suggested that he should do the same thing, but focus on mental disorder instead (Faris, 1998).^13^

Although many female graduate students studied at the Chicago School during the 1920s and 1930s, Cavan was one of the few to gain the recognition that many of her male counterparts enjoyed. Nevertheless, her reputation tended to be based on her skill as a writer of textbooks, rather than a producer of original research. It has been argued that this was partly because she left the University of Chicago for the more inconspicuous Rockford College, where her husband, Jordan Cavan, was a professor (Moyer, 1989: 173). There she was unable to attract graduate students who would have given her a more direct legacy. However, associating Cavan only with her textbooks is both unfair and misleading, given the influence of her monograph *Suicide*, one of the first English language studies of the subject following Durkheim’s (1897) classic, as well as her subsequent research on criminology (Moyer, 1989).^14^ Cavan also wrote an article on the history of the Chicago School, which succinctly provides many insights into the approach of the School and what it was like to be a graduate student there (Cavan, 1983).

Ruth Shonle grew up in Tuscola, Illinois, and her original career plan was to train to be a teacher, the only profession she recognised as being open to women. Her parents lacked the money to send her to college, so she took an office job to save money, attending business school in the evenings. She eventually earned enough money to transfer to the University of Chicago in 1920, undertaking undergraduate studies in English and economics and then a Master’s degree in sociology. Her MA thesis (1923) was on the Oneida community and the Mormons, which generated her first four articles. Her decision to turn to suicide for her doctoral research was – as with most Chicago School graduate students – suggested by her supervisor, in this case Ellsworth Faris (Moyer, 1989: 178). This doctoral project, finished in 1926, would lead to her monograph, *Suicide*, which, according to Robert Faris (1967/1970: 84), was completed while she was working as a department secretary at the university, a rather impressive feat in itself.

Unlike *Mental Disorders in Urban Areas*, which focused squarely on the geographical distribution of mental disorder in Chicago (as well as a comparative chapter on Providence, Rhode Island), Cavan’s (1928) monograph addressed the issue of suicide from a variety of perspectives. She began by examining suicide in Europe and the USA in historical and contemporary perspectives before briefly exploring the phenomenon in indigenous groups in various parts of the world, as well as India, China and Japan. Then, she turned to the situation in Chicago, using coroners’ records (over 1000 cases between 1921 and 1923), United Charity records, diaries (one chapter was entitled ‘The diaries of two suicides’), life histories and newspaper reports. In particular, she analysed the 391 cases reported in 1923, noting whether there were underlying psychiatric problems and providing in-depth studies of individual cases.^15^ Finally, she used maps, not only to demonstrate the distribution of suicides across Chicago but also to compare the suicide rates to those of divorce, murder, alcoholism and ‘other indications of disorganization’ (p. 101). As Shaw would show with respect to delinquency, and as Faris and Dunham would show with respect to mental disorder, there was a distinct pattern of the areas where suicides occurred in the city. Specifically, Chicago had a ‘suicide belt’, encompassing: (1) the Loop, or central business district, characterised by ‘cheap hotels for men and sooty flats over stores’; (2) the Lower North Side, including its ‘shifting population of unattached men and an equally shifting population of young men and women in the rooming house areas’; (3) the Near South Side, which linked the Loop to the Black Belt; and (4) the West Madison area or Hobohemia, ‘with its womanless street of flophouses, missions, cheap restaurants, and hundreds of men who drift in aimless, bleary-eyed abandon’ (p. 81). In contrast, other areas of Chicago had very few suicides.

Cavan’s analysis of these suicide belt communities revealed that suicide was linked to social disintegration and low socio-economic status. When people lacked a ‘community life’ and were restless, they were at risk of ‘personal disorganization’ and dissatisfaction which, in turn, increased the probability of suicide (pp. 103–4). As with many of the social psychiatrists who would pursue similar lines of inquiry, Cavan emphasised the potential for people in such circumstances to succumb to ‘forbidden stimulations and pleasures’, in other words, moral vices, rather than the damaging effects of poverty and inequality (p. 104). Disorganised communities were unable to enforce the sort of social controls to keep such behaviours in check. Foreshadowing the debates about ‘downward drift’ that bedevilled Faris and Dunham (that is, people with pre-existing mental disorders drifted into places such as the Loop and Hobohemia), Cavan added that such areas also attracted people who balked at the social controls typically present in more integrated communities, particularly smaller towns or rural environs.^16^ As Mical Raz (2013) has argued persuasively with respect to the War on Poverty during the 1960s, this tendency to focus on the moral failings of the poor, and especially Black Americans, led to programmes intended to effectively ‘teach’ the poor how not to be poor, rather than give them the material resources required to lift them out of poverty or tackle racism and inequality more directly.

In his introduction to *Suicide*, Ellsworth Faris urged that sociological studies of suicide could provide the basis for a ‘program of control’ (Cavan, 1928: xvii). Fittingly, Cavan concluded her book with some thoughts on how to ‘control’ suicide. Here, too, she strayed away from recommending any socio-economic changes and concentrated on the importance of changing societal attitudes and individual behaviour. Perhaps reflecting on her study of suicide in indigenous communities as well as the role of religion in discouraging suicide, Cavan suggested that by making suicide ‘repulsive enough [it] would not occur so frequently’, an argument quite in contrast with today’s focus on destigmatisation (p. 332). She also advised training individuals to become more ‘reflective’ and ‘resourceful’ and advocated early diagnosis and treatment, which was happening in Chicago’s many mental hygiene and child guidance clinics (pp. 332–3). But she said little about how to alter the disorganisation in many of Chicago’s communities which provided the soil in which suicidal thoughts could root. Although Chicago School sociologists helped to establish the idea that it was the environment, not the individual and their hereditary make-up, that mattered most in understanding the root causes of mental disorder and other social problems, they – along with the social psychiatrists who followed them – tended to shy away from demanding social change.

## Conclusion

In some ways, Faris and Dunham’s *Mental Disorders in Urban Areas* was less ambitious than many of the pioneering Chicago School studies that preceded it. Chiefly, its focus on providing the geographical distribution of mental disorder in Chicago meant that it lacked the qualitative methods introduced by Anderson, let alone the combination of both quantitative and qualitative methods that characterised the works of Shaw and Cavan. Faris and Dunham’s conclusions reflected ideas of community disintegration, much like their predecessors, and they were not particularly interested in the clinical sociology of Wirth and Burgess or the activism of Shaw. But it is possible that their methodical, painstaking approach to plotting, depicting and explaining psychiatric epidemiology in Chicago was one of the reasons that their study became so influential in the decades that followed. The well-funded social psychiatry projects in the 1950s (some boasting nearly 100 staff) may have had the resources to do much more than Faris and Dunham (two graduate students, it must be emphasised), but their task was essentially the same: to understand the link between the socio-economic environment and mental health.

The other vital contribution of Faris and Dunham, as well as their Chicago School predecessors, was to show that the insights of sociologists, as well as anthropologists, other social scientists and even historians, were essential to understanding psychiatric epidemiology. The social psychiatry studies that emerged after World War II are notable for their interdisciplinarity. While some, such as the New Haven Study, were led by psychiatrist–social scientist partnerships, other social psychiatrists, such as the Leightons of the Stirling County Study, were qualified in both psychiatry and the social sciences. Moreover, all the major social psychiatry studies of the post-war period were historically grounded, often providing lengthy accounts of the history of the areas studied in their publications. Assessed from the standards of today, some aspects of these social psychiatry studies can frustrate – for example, they tended to blame the poor for their lot and were hesitant to demand social change – but their genuine interdisciplinarity is something from which scholars seeking to understand psychiatric epidemiology today could certainly learn. This is particularly true with respect to deriving and delivering the sort of progressive policies required in order to tackle the socio-economic problems that researchers have associated with mental illness since the 1920s.^17^ Above all, the Chicago School demonstrated that context matters enormously in any attempt to grapple with all social problems, especially mental illness.

## Notes

1. The Chicago School of Sociology was not alone in influencing psychiatry. Other ground-breaking social science departments, such as anthropology at Columbia and sociology at Yale, also helped to shape psychiatric understanding, but for psychiatric epidemiology the Chicago School was arguably the first, the most active and the most influential. There have been a number of histories of the Chicago School of Sociology, including one by Robert EL Faris himself (Faris, 1967; see also: Carey, 1975; Harvey, 1986; Kutz, 1984). Arguably, the most complete is Jean-Michel Chapoulie’s *Chicago Sociology*, which was translated into English in 2020.

2. According to many contemporary observers, the term ‘the Chicago School’ was not coined by sociologists in Chicago, but by others. It was being used, however, as early as 1930 (Cavan, 1983: 407–20).

3. Unlike most monographs to come out of the Chicago School, *The Polish Peasant* was not published by the University of Chicago Press. This was because Thomas was caught up in a sex scandal in 1918, which cost him his job at the University of Chicago and led to the publisher cancelling the publication contract. After his dismissal, he was paid by Chicago philanthropist Ethel Dummer (1866–1954) to research female prostitutes and unwed mothers, publishing this as *The Unadjusted Girl* in 1923.

4. Anthropology had always been the junior party in the department, usually boasting only one member of staff. It eventually split from sociology and formed its own department in 1929 (Cavan, 1983: 410).

5. Among the psychiatrists influenced by Park and Burgess was Adolf Meyer (1866–1950), arguably the most influential psychiatrist in the USA during the first half of the twentieth century and a key figure in the mental hygiene movement (March and Oppenheimer, 2014).

6. Permission to publish Figures 2, 3 and 4 granted by John H Faris.

7. Following his graduate studies, Anderson also had a peripatetic career that saw him work in the civil service, with the State Department in Germany and in academia in Canada (Iverson, 2009: 181–3).

8. The Laura Spelman Rockefeller Fund provided funding to publish many Chicago School graduate studies with the University of Chicago Press between 1923 and 1930. The conclusion of this scheme coincided with the Chicago School’s declining influence within sociology (Cavan, 1983: 417).

9. Later, Robert Faris (1967: 66) claimed that *The Hobo* used ‘no formal research technique and yielded no new sociological principles’. Anderson himself admitted that *The Hobo* provided little in the way of sociological theory, but given the degree to which participant observation became a staple sociological methodology, Faris’s account says more about how he and other Chicago School insiders felt about Anderson, his unconventional background and his research subject: the hobo. Indeed, Anderson went to New York University for doctoral studies, partly to escape such dismissive attitudes (Rauty, 1998).

10. Anderson concluded the book with an Appendix that outlined a detailed list of both national and local recommendations.

11. Faris and Dunham (1939: xxi) also note in the preface to their book that ‘Mrs. Clifford R. Shaw’ had done a ‘preliminary study of the distribution of cases of mental disorder admitted to the Cook County Psychopathic Hospital’ in 1928, which gave their study a kick-start. I have failed to find anything more, however, about Clifford Shaw’s wife.

12. During an era when eugenic theories were influential, the boldness of such conclusions should not be underestimated (Dowbiggin, 1997: 128–9, 132).

13. In Robert Faris’s history of the Chicago School, he added that *Suicide* was ‘one of the most important studies of disorganization’ (Faris, 1967: 84).

14. In 1997, however, the American Society of Criminology established an award for young scholars in Cavan’s honour. Interestingly, Cavan only mentions Émile Durkheim’s *Le suicide* (1897) in the context of debates about the relationship between alcoholism and suicide. Many of the Chicago School monographs are notable for their rather sparse referencing, but Durkheim’s relative absence is notable and perhaps indicates a desire to look to US, rather than European, scholarly influences.

15. The figure is listed as 291 on page 112 of *Suicide*, but 391 elsewhere in the book.

16. Regarding the role of urban life, Cavan was fairly equivocal overall. Cities appeared to be more conducive to suicide than smaller communities, but they brought great benefits to most people.

17. For example, I have recently made the case for Universal Basic Income as a preventive approach to mental illness (Smith, 2023: Epilogue).
